# Multinomial tree models for assessing the status of the reference in studies of the accuracy of tools for binary classification

**DOI:** 10.3389/fpsyg.2013.00694

**Published:** 2013-10-03

**Authors:** Juan Botella, Huiling Huang, Manuel Suero

**Affiliations:** Department of Social Psychology and Research Methods, Facultad de Psicología, Universidad Autónoma de MadridMadrid, Spain

**Keywords:** binary classification, gold standard, multinomial tree models, imperfect reference, diagnostic accuracy

## Abstract

Studies that evaluate the accuracy of binary classification tools are needed. Such studies provide 2 × 2 cross-classifications of test outcomes and the categories according to an unquestionable reference (or gold standard). However, sometimes a suboptimal reliability reference is employed. Several methods have been proposed to deal with studies where the observations are cross-classified with an imperfect reference. These methods require that the status of the reference, as a gold standard or as an imperfect reference, is known. In this paper a procedure for determining whether it is appropriate to maintain the assumption that the reference is a gold standard or an imperfect reference, is proposed. This procedure fits two nested multinomial tree models, and assesses and compares their absolute and incremental fit. Its implementation requires the availability of the results of several independent studies. These should be carried out using similar designs to provide frequencies of cross-classification between a test and the reference under investigation. The procedure is applied in two examples with real data.

## Introduction

Tools for binary classification are regularly used in psychology, as in screening processes for the early detection of certain disorders or risk factors for such disorders. Their objective is to detect a specific status and assist in the decision-making process. Procedures that are able to identify a specific status quite accurately are available, but they are expensive and consequently large scale applications are unfeasible. Therefore, psychologists and other health professionals are looking for alternative classification tools which are simple, effective, and inexpensive. Often these alternatives are questionnaires that contain multiple items, the scores of which generate a dichotomy based on a simple rule that the practice suggests as an effective screening. For example, it has been proposed to use the Alcohol Use Disorders Identification Test (AUDIT) with a cut-off of *X* ≥ 8 for detecting alcohol-use disorders (Babor et al., 2001) or the Mini-Mental State Examination (MMSE; Folstein et al., 1975) with a cut-off of *X* ≥ 24 to detect dementia.

**Figure 1:**
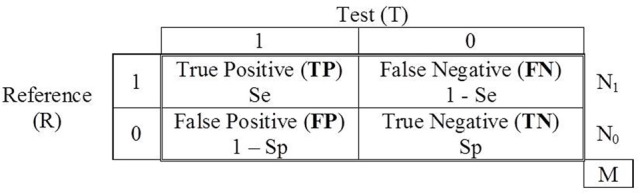
**Contingency table of the binary classifications of a reference (R) and a test (T)**.

The effectiveness of those tools is estimated by studies that assess their accuracy in classification. These studies require a previous classification by a reference (R) that is considered unquestionable and which provides the true status of each participant. The results are summarized in 2 × 2 tables with the frequency of participants that are positive for the condition sought (denoted by 1) and of those who are not (denoted by 0) according to R, crossed with the result (positive or negative) of the test (T) for which the diagnostic accuracy is going to be assessed. For example, if the AUDIT test is applied, respectively, to groups of individuals showing alcohol abuse (N_1_) and without alcohol abuse (N_0_), the status is crossed with the binary classification by the test, and a table similar to that in Figure 1 is generated. The table represents the scenario of evaluation, where the joint frequencies of the results of R and T are summarized. The four events are represented as TP (true positives), FN (false negatives), FP (false positives), and TN (true negatives). The sum of the four frequencies equals the total sample size of participants (M). The empirical prevalence in the study is the proportion of participants with the status “1” in the study, (*TP* + *FN*)/*M* = *N*_1_/*M*.

The diagnostic accuracy of a binary classification test can be summarized by two probabilities: the probability of a positive result given the status “1,” *P*(*T* = 1|*S* = 1), and the probability of a negative result given the status “0,” *P*(*T* = 0|*S* = 0). In a perfect performance test both probabilities will equal 1 and would provide a contingency table where *FN* = *FP* = 0. However, in practice, the tests for which the accuracy is assessed have suboptimal reliability, and the probability *P*(*T* = 1|*S* = 1), known as the *sensitivity* of the test, will be less than 1. Similarly, the probability *P*(*T* = 0|*S* = 0), known as the *specificity* of the test, will also be less than 1. The sensitivity and specificity of the test T are denoted here by *Se*_*T*_ and *Sp*_*T*_, respectively.

## When the reference is not a gold standard

The traditional design of studies evaluating the accuracy of screening tests implies that R is a device of perfect accuracy; that is why it is called *gold standard* (GS). However, sometimes R also has a suboptimal reliability and is therefore not a gold standard. In these cases it is referred to as an *imperfect reference* (IR). Some authors have highlighted that difficulties arise when the reference is imperfect, the most important being that the estimates of sensitivity and specificity are biased, as is the calculation of the prevalence (e.g., Valenstein, 1990). With regard to the prevalence, if the study is performed with an IR, the difference between the *observed* (or apparent) *prevalence*, obtained from the frequencies in Figure 1 and defined above as (*TP* + *FN*)/*M*, and the *empirical prevalence* (which is estimated to fit the models) must be explicitly highlighted. When the reference is imperfect the observed prevalence is calculated from “contaminated” frequencies, whereas the empirical prevalence is the (unknown) actual proportion of targets in the study.

When the reference is a GS the observed prevalence and the empirical prevalence are identical, but when it is an IR they may be very different. By fitting IR models the parameter reflecting the prevalence, π, can be estimated to give an approximation to the empirical prevalence of the study (the actual proportion of participants with status “1”). The use of an IR allows some traffic of counts between FP and TP on the one hand, and TN and FN on the other. This traffic generates the difference between the empirical prevalence and the observed prevalence. Some solutions have been proposed to improve the performance of studies when R is imperfect (Rutjes et al., 2007; Reitsma et al., 2009; Trikalinos and Balion, 2012). However, before you can apply them it is necessary to assume whether R is flawed or not (whether R shows sensitivity and/or specificity lower than 100%).

Our main objective is to propose a procedure to help with the choice between two different scenarios, related to the status of a reference as a GS or an IR. The procedure consists of fitting two nested multinomial tree models (MTM) and assessing their goodness-of-fit.

## Multinomial tree models

The MTM employed here is imported from the general framework of the multinomial processing tree models. This class of models is widely used in psychology for the study of cognitive processes (Batchelder, 1998; Batchelder and Riefer, 1999; Erdfelder et al., 2009). In the present application, cognitive processes (the discrete cognitive states generated by those processes, or the responses generated by those processes) are substituted by the result of administering two tools for categorizing the status of the participants in a given study.

We have used MultiTree (Moshagen, 2010), a software specifically developed for that class of models. Parameter estimation proceeds by employing the expectation maximization (EM) algorithm (Hu and Batchelder, 1994). Several statistics are available for assessing both the absolute and incremental fitting of the models (García-Pérez, 1994; Hu and Batchelder, 1994). For the goodness-of-fit test of a model, the degrees of freedom are defined as the difference between the number of independent data categories and the number of parameters estimated. For the incremental fitting, the degrees of freedom are the difference between the degrees of freedom of the two models being compared. The pattern of results of the goodness-of-fit tests for the absolute fit of both models and the incremental fitting, as nested models, will be the basis for selecting one of the models (Moshagen and Hilbig, 2011).

Given a single data set, several of the models considered here are not identifiable, and/or, testable. The reason is that the number of estimated parameters exceeds (or is equal to) the number of independent categories. The solution we propose to this problem can only be applied if some minimum number of independent and homogeneous studies that provide data regarding the classification of the test (and have used the same reference) are available. It is assumed that all those studies share the same parameters of accuracy, but each has its own parameter of prevalence. In the two examples with real data described below four independent studies are employed.

## Models of assessment

Two models of assessment are involved in the present research. In the assessment scenario involved in *model 1* the reference is a GS, whereas in *model 2* it is an IR.

### Model 1: the reference is a gold standard

**Figure 2:**
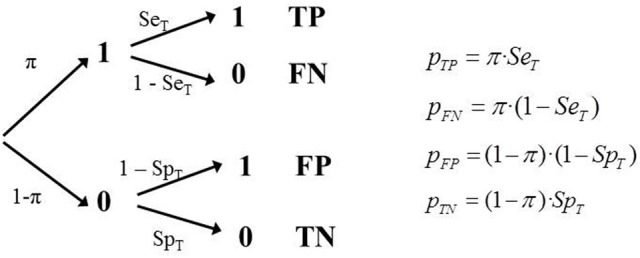
**Tree diagram of Model 1 (GS) and the corresponding equations**.

The study assesses the diagnostic accuracy of a test where the sampling model generates a parametric prevalence (proportion of participants with status “1” in the population, according to the study's sampling model) equal to π. The process of assessment can be represented by a tree diagram as in Figure 2.

Although it is possible to fit the model with only one study, testing the model requires more than one independent study that provides data regarding the classification test (and have used the same reference). A study provides three independent frequencies (the fourth is determined by M) therefore to fit the model three parameters must be estimated. And consequently if we do so there would be no degrees of freedom available to test it. However, if there are several independent studies using the same classification tool, and the same criterion, it is possible to fit and test the model properly, assuming that the sensitivity and specificity are independent of the study's empirical prevalence.

It is assumed that results are available from a set of *k* independent studies, each with a different sampling model, and therefore with a different empirical prevalence, π_*i*_; in each study there are different total sample sizes. Each study provides four frequencies with three degrees of freedom. The number of independent parameters to be estimated is *k* + 2. The number of degrees of freedom is 3*k* − (*k* + 2) = 2*k* − 2. Consequently, the minimum number of studies required to test this model is 2 because then the parameters will be two π values plus *Se*_*T*_ and *Sp*_*T*_. The degrees of freedom would be 2. It is defined a separate tree for each study and a simultaneous fit is performed. Naturally, the estimate would be more reliable the larger the number of independent estimates (*k*).

### Model 2: the reference is imperfect

Sometimes the reference employed in assessment studies is not a real GS (the sensitivity and/or specificity of R, denoted by *Se*_*R*_ and *Sp*_*R*_, are less than 1). In psychology, categorical diagnostic assessment is often performed using an in-depth interview where the DSM criteria (American Psychiatric Association, 2002) are checked, combined with complementary tests. This is usually considered an almost perfect reference (a GS, in practical terms). However, in some studies assessing the diagnostic capability of binary classification tools, the diagnosis employed as the reference is not done like this. Instead, it is done with another psychometric test with relatively good properties, but with sub optimal reliability. For example, to assess the accuracy of SCOFF (S*ick*-C*ontrol*-O*ne*-F*at*-F*ood*; Morgan et al., 1999; Hill et al., 2010), the *Eating Attitudes Test* (*EAT*; Garner et al., 1982) is sometimes used as the reference. In the “short” version of this test (26 items scored 0–3) a cut-off of *X* = 20 is often used for classification (e.g., Berger et al., 2011). Although errors may occur in a diagnostic interview its reliability is undoubtedly higher than that of a test such as *EAT*.

The main difference between model 1 and model 2 is that in model 1 the observed prevalence of the study, (*TP* + *FN*)/*M*, is the true empirical prevalence. By contrast, in the scenario implied in model 2 the observed prevalence does not correspond to the true empirical prevalence in the study. In the TP and FN cells there is an unknown fraction of observations that are not real 1s, and/or in the FP and TN cells there is an unknown fraction of observations that are not real zeros.

**Figure 3:**
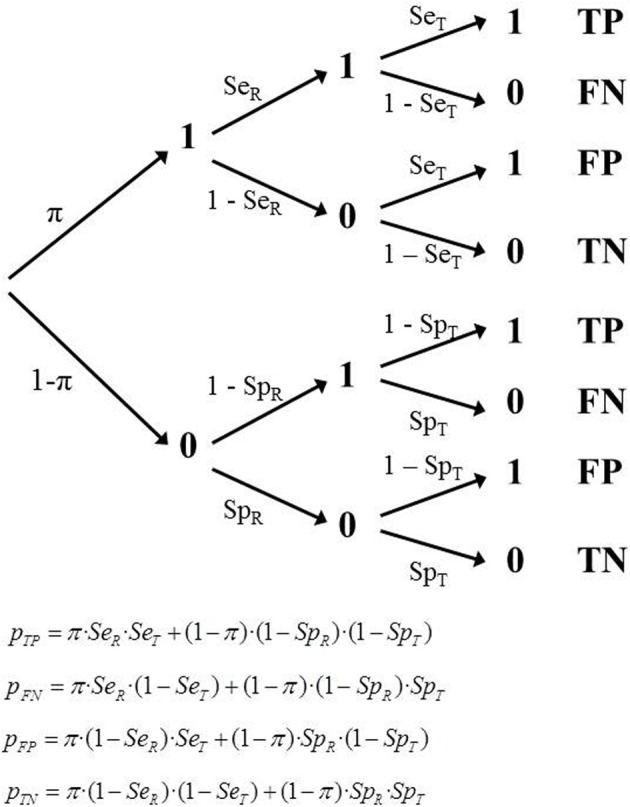
**Tree diagram of Model 2 (IR) and the corresponding equations**.

Consequently the tree model must incorporate new features, represented by new parameters. Specifically, it must include two values of sensitivity (*Se*_*R*_, *Se*_*T*_) and two values of specificity (*Sp*_*R*_, *Sp*_*T*_). This means that some observations that are categorized as TP should actually have been coded as FP, since R had misclassified them as 1 when they were 0. The same is true in the remaining cells. Each of the four observed frequencies are composed of a genuine part of observations, plus a portion that have been counted in the wrong cell, because they were incorrectly classified by R (as 1, when they were a 0, or vice versa; Figure 3).

When referring to a single study, this model includes five parameters (π, *Se*_*T*_, *Sp*_*T*_, *Se*_*R*_, *Sp*_*R*_), but there are only three degrees of freedom available. The model cannot be identified or tested. However, if there are several studies with independent estimates then it is possible to properly estimate the parameters. As in the previous model, we assume a set of *k* independent studies, each with a different sampling model and therefore with a different empirical prevalence, π_*i*_. The number of independent parameters to be estimated is *k* + 4. The number of degrees of freedom is 2*k* − 4. The minimum number of studies required to fit and test this model is 3, because the parameters are three values of π, plus the two sensitivities and two specificities. The degrees of freedom would be 2.

Although model 2 is appropriate in many situations, it makes a debatable assumption about the independence between the classifications provided by R and T within each category. Thus, it assumes that the probability that the test will yield a positive result when applied to a participant with status “1” remains the same, regardless of whether this case has given a positive or negative result in R. However, a refinement of model 2 that relaxes the assumption of independence between the classifications provided by R and T is not identifiable within the present framework and is not discussed in this paper.

## Assessing and selecting a model

A model's fit in MTM is usually assessed by the likelihood Ratio Test through the *G*^2^ statistic (Read and Cressie, 1988), asymptotically distributed as χ^2^ when the model is true. This statistic allows the decision about whether the model must be rejected or not. But it is possible for more than one model to fit, so the problem of selecting a particular model arises.

If a model has a significantly lower discrepancy it is considered to be a better representation of what is being modeled (Ulrich, 2009). However, it is well-known that more complex models (for example, those with more free parameters) tend to fit better, because they have more flexibility and can capitalize on chance (Pitt et al., 2002). The different levels of complexity must be considered when comparing models. In our context GS and IR are nested models (GS is a particular case of IR, with *Se*_*R*_ = Sp_R_ = 1). When both models fit the data well the difference between their *G*^2^ statistics is asymptotically distributed as χ^2^ with two degrees of freedom. So, the incremental fitting can also be tested.

Several criteria and indices have been proposed that essentially reflect the trade-off between the predictive accuracy of the model and the model's complexity. The Akaike (1974) and Bayesian (Schwartz, 1978) criteria have been criticized because they do not fully account for all relevant dimensions of complexity. However, an interesting alternative is the Minimum Description Length criterion, which has recently been proposed for selecting MTM models (Wu et al., 2010). When two models have the same fit the MDL selects the less complex, as the Akaike and Bayesian criteria do. But the MDL also takes into account other dimensions of the models' complexity, such as their functional form. This criterion as implemented in MultiTree (Moshagen, 2010) will be employed in the examples below.

For both GS and IR models, when fitted as in the two examples below (with four independent studies), the rank of the Jacobian matrix is not lower than the number of parameters estimated. Thus, the models are locally identifiable. However, as is shown in the examples, model 2 is always associated with two different sets of estimates, as it shows a problem of global identifiability. When IR models are fitted in repeated occasions on the same data MultiTree provides alternatively two sets of parameters that yield the same (and minimum) value for *G*^2^. Thus, using the data in the two examples below, different runs can produce any of the two sets of parameters in Table 1. In each example the specificity in set 2 is the complementary of the sensitivity of set 1 and the sensitivity in set 2 is the complementary of the specificity of set 1. This happens both in the reference and the test.

**Table 1:** **Parameters estimated in the two solutions provided by Multitree for Model 2, IR, in the two examples with real data**.

		***Se*_R_**	***Sp*_R_**	***Se*_T_**	***Sp*_T_**	***G*^2^**
AUDIT	Set 1	0.996	1.00	0.637	0.960	13.985
	Set 2	0.000	0.004	0.040	0.363	
MMSE	Set 1	0.876	1.00	0.864	0.872	12.136
	Set 2	0.000	0.124	0.128	0.136	

It could be argued that there is no means to judge which set of estimates is correct. Although in some special cases could be difficult, in most cases it is a matter of common-sense. Both the reference and the test are chosen for the study because they are well-known to be effective. The purpose of the study is to obtain an accurate estimation of the efficacy of the test (and the experience suggests that it works better than tossing a coin). As one of the two sets of parameters involves a less than random performance, the other must be chosen. Even worst, the values in set 2 reflect accurate but perverse tools. They reflect a test which classifies the vast majority of targets as normal and vice versa. So, one of the sets is congruent with the estimates provided by the individual studies while the other is incongruent with them (see the examples below). Nevertheless, global identifiability can be achieved by imposing parametric order constraints (see Knapp and Batchelder, 2004). If when running with MultiTree both examples of Table 1 it is imposed the constraint that the four parameters reflecting accuracy (*Se*_*T*_, *Sp*_*T*_, *Se*_*R*_, *Sp*_*R*_) are higher than 0.50 then the correct solution is always reached. However, there could be other types of tests or measurement contexts in which the choice is not as clear as in our examples. Thus, it is possible that only three of those parameters are higher than 0.50. For example, sometimes the target status is difficult to detect by the reference (*Se*_*R*_ around 0.50) but the study is still worthy because specificity is very high. In any study of this type the two solutions must be obtained and compared. It is possible that in none of the two solutions the four parameters are simultaneously higher than 0.50; in those cases other models of measurement are probably needed.

## Two examples with real data

Applying the procedure requires that the *k* studies included are homogeneous. This is not a problem in simulation studies, but with real data it is impossible to have studies that are exact replicates. Real data differ in such characteristics as the type of professional who manages R and T, the context in which it is applied, or the language version employed in the test. However, within certain boundaries the studies can and should be homogeneous, enabling the interpretation and recognition of the estimated parameters in all of them. The main objective here—to determine whether or not the reference is a GS—can be addressed with a level of homogeneity that need not be completely strict. However, as a consequence of not using exact replicates some additional misfit must be expected. This is the rationale for preferring among the conventional alpha levels the more liberal for our purposes (0.01 instead of 0.05) when testing the absolute fit of the models. However, power analyses will be performed for every test.

### The test AUDIT and self-report of drinking as a reference

We used four independent studies to assess the accuracy for the classification of the *Alcohol Use Disorder Identification Test* (*AUDIT*; Babor et al., 2001). These studies shared the same specific target population, the elderly, and used the same reference for the classification. The reference is an objective (although self-reported) amount of alcohol consumption of at least 14 drinks per week. The test is employed with a cut-off value of *X* ≥ 8 as a rule for the binary classification in males. The top panel of Table 2 identifies the studies. It also shows the raw data, the sensitivity, the specificity, and the observed prevalence in each study.

**Table 2:** **Raw frequencies of the primary studies included in the two examples with real data**.

**Test**	**Study**	***TP***	***FN***	***FP***	***TN***	***Se***	***Sp***	**Prev.**
AUDIT	Bradley et al., 1998	58	47	6	150	0.552	0.962	0.402
	Philpot et al., 2003	13	4	7	104	0.765	0.937	0.133
	Reid et al., 2003, (sample 1)	22	3	6	148	0.880	0.961	0.140
	Reid et al., 2003, (sample 2)	7	3	8	243	0.700	0.968	0.038
MMSE	Brayne and Calloway, 1989	24	5	31	205	0.828	0.869	0.109
	Brodaty et al., 2002	66	16	48	153	0.805	0.761	0.290
	Clarke et al., 1991	137	17	28	122	0.890	0.813	0.507
	Cullen et al., 2005	40	4	138	933	0.909	0.871	0.039

The two top rows of Table 3 show some of the results provided by MultiTree (Moshagen, 2010) when fitting the two models. There are several reasons for choosing model 1 as the one which better describes the behavior of the reference and the test in this example. Firstly, the goodness-of-fit statistic for the GS model shows an acceptable value (*p* > 0.01). When two more parameters are included (model 2) the fit does not improve significantly (the statistic is virtually equal, but with two more parameters). Secondly, in model 2, the values for *Se*_*R*_ and *Sp*_*R*_ are both close to 1 (rounding to the third decimal gives 0.996 and 1.0, respectively), the values representing an optimal reference or GS. Thirdly, the criterion employed for model selection, MDL, gives a smaller value for the GS model than for the IR model (see the last column in Table 3).

**Table 3:** **Parameter estimates, goodness-of-fit and Minimum Description Length of the two models for the AUDIT and the MMSE data**.

**Test**	**Model**	**Reference**				**Test**				**Goodness of fit**			***C*_FIA_**	***MDL***
		***Se*_R_**		***Sp*_R_**		***Se*_T_**		***Sp*_T_**		***G*^2^**	***df***	***p***		
		**Estim**	***SE***	**Estim**	***SE***	**Estim**	***SE***	**Estim**	***SE***				
AUDIT	1—GS	–		–		0.637	0.038	0.960	0.008	13.99	6	0.030	17.9	574.8
	2—IR	0.996	0.054	1.00	0.011	0.637	0.044	0.960	0.011	13.99	4	0.007	20.1	577.0
MMSE	1—GS	–		–		0.864	0.020	0.852	0.009	21.14	6	0.002	20.1	1495.2
	2—IR	0.876	0.040	1.00	0.004	0.864	0.023	0.872	0.011	12.14	4	0.016	23.0	1493.6

We have also performed a power analysis (*post-hoc* analysis; Faul et al., 2007) of the test for the incremental fit, as follows. In the nested model (GS) the parameters describing the reference are *Se*_*R*_ = Sp_R_ = 1. We have established that for the test being convincing it must have enough power to detect a small-medium effect size in the sense of Cohen (1988; *w* = 0.10 is considered small and *w* = 0.30 is considered medium). In this case the small effect size is obtained (approximately) by setting in the alternative model the values *Se*_*R*_ = Sp_R_ = 0.95 (the effect size is *w* = 0.097). With alpha 0.05 the power is 0.707. The small-medium effect size is obtained (approximately) by setting the values *Se*_*R*_ = Sp_R_ = 0.80 (the effect size is *w* = 0.20081). With alpha 0.05 the power is 0.9997. When alpha is set at 0.01 the power values for the same effect size values are 0.475 (for small w) and 0.998 (for small-medium w).

In summary, the model chosen was model 1, which implies that the reference employed (self-report of at least 14 drinks per week) works most probably as a GS. The best estimates of the sensitivity and specificity of the test are the values obtained using model 1 (*Se*_*T*_ = 0.637; *Sp*_*T*_ = 0.960). The results of these four studies suggest that whereas the AUDIT almost never classifies a normal behavior as a disorder, (about 4%), it misses a considerable number of alcohol abuse problems in the elderly (slightly more than one-third).

The set of parameters in Table 3 is the one obtained after imposing the parameter constraints described above (*Se*_*T*_, *Sp*_*T*_, *Se*_*R*_, *Sp*_*R*_ > 0.50). Common-sense also advises the same conclusion, as examining the calculated sensitivities and specificities of the studies (Table 2) it is clear that only this solution is congruent with the meaning of the parameters.

### The test MMSE and the CAMDEX as a reference

We also used four independent studies that provide data allowing the assessment of the accuracy for the classification of the *Mini Mental State Examination* (*MMSE*; Folstein et al., 1975). This is a test for detecting unspecific dementia, although it is often used to detect the early stages of Alzheimer's disease. The test is employed with a rule for classification based on a cut-off value of *X* = 24. The reference employed in the primary studies included the *Cambridge Mental Disorders of the Elderly Examination* (*CAMDEX*; Roth et al., 1986). The bottom panel of Table 2 identifies the studies and shows the raw data, the sensitivity, the specificity, and the observed prevalence in each study.

Table 3 (two bottom rows) shows the results provided by MultiTree. There are several reasons for choosing here model 2 as the one that better describes the behavior of the reference and the test. Firstly, the goodness-of-fit for model 1 shows a significant deviation between the empirical and predicted frequencies (*p* < 0.01), but model 2 does not (*p* > 0.01). Secondly, although the value for *Sp*_*R*_ in model 2 is virtually 1 (the optimal classification of “normals”), the value of *Se*_*R*_ is far from its upper boundary. In this particular example, the parameters of model 2 indicate that the reference has virtually perfect specificity, whereas the sensitivity is suboptimal (*Se*_*R*_ = 0.876). Assuming this value for the sensitivity of the reference, the estimated parameters for the MMSE are *Se*_*T*_ = 0.864 and *Sp*_*T*_ = 0.872, respectively. Thirdly, the criterion employed for model selection, MDL, provides a smaller value for the IR model than for the GS model (Table 3).

A power analysis of the test for the incremental fit test similar to that of the AUDIT yielded the following results. In this case, to achieve a small effect size (*w* = 0.099) the values in the alternative model should be set as *Se*_*R*_ = Sp_R_ = 0.975. With alpha 0.05 the power is 0.982. The small-medium effect size is obtained (approximately) by setting the values *Se*_*R*_ = Sp_R_ = 0.91 (the effect size is *w* = 0.20313). With alpha 0.05 the power is 1 when rounded to three decimals. When alpha is set at 0.01 the power values for the same effect size values are 0.932 (for small w) and 1 (for small-medium w).

As with AUDIT, common sense advises choosing the set 1 in Table 1 rather than set 2, as the only one that is congruent with the meaning of the parameters. The same set is the obtained after imposing the same constraints as in the AUDIT example. In summary, the model chosen for the MMSE was model 2, which implies that the reference employed in this set of studies (CAMDEX) is most probably an IR. Then, if the accuracy of the MMSE is assessed without acknowledging that the reference has suboptimal reliability, then the estimate of the accuracy of the test is biased.

The results of the example with the MMSE test can give the impression that in the end the difference is small and not worth the effort to study and assess the accuracy of the reference. Consider a numerical example with more dramatic results. Suppose the test X has *Se*_*X*_ = 0.90 and *Sp*_*X*_ = 0.85 and the accuracy is evaluated using a gold standard reference. If there are 200 target cases in the study and 800 normal cases (observed prevalence = empirical prevalence = 0.20) the expected frequencies are 180 (TP), 20 (FN), 120 (FP), and 680 (TN). However, if in the same study the reference is imperfect and has *Se*_*R*_ = 0.90 and *Sp*_*R*_ = 0.90 then the expected frequencies are 174 (TP), 86 (FN), 126 (FP), and 614 (TN). As a consequence, if the imperfection of the reference is not acknowledged the expected estimates of the sensitivity and specificity are 0.669 [calculated as 174/(174 + 86)] and 0.830 [calculated as [614/(614 + 126)], respectively, instead of the actual 0.90 and 0.85 values. Furthermore, although the empirical prevalence is 0.20 the observed prevalence is now 0.26 [calculated as (614 + 126)/1000].

In short, if researchers do not recognize the imperfection of the reference they will not be aware that dozens of cases have been misclassified in his/her data, and will report underestimations of the accuracy of the test. Furthermore, this could lead to unsatisfactory choice when several tests are available as candidates for the same diagnostic purpose, as decision is based on comparisons of their accuracies. An apparently better test would be preferred only because its diagnostic accuracy has been assessed against a gold standard reference. Other equally or even more suitable tests would be eliminated because they have been validated using imperfect references. The choice of a test for the screening process will be flawed.

## Discussion

The reliability of the reference must be evaluated and taken into account when assessing the accuracy of tools for binary classification in screening processes (Valenstein, 1990). Whilst in most statistical models it is assumed that the reference is perfectly reliable (GS), in psychology the references usually employed are suboptimal. In medicine, the references are sometimes objective states that can be checked with almost perfect accuracy. But in psychology we often lack such references. Construct validity is an enduring concern for researchers in psychology (Cook and Campbell, 1979; Messik, 1989, 1995), because we know that the references are almost always suboptimal. In some cases the accuracy of the reference is so high that it can be taken as a GS without having any relevant impact on the results of the estimates. However, as a general rule their status should be assessed.

Sometimes it is acknowledged that the reference used in studies that assess the accuracy of screening tools may be imperfect. Procedures have been proposed to account for this suboptimal reliability in estimating the accuracy of the test (Rutjes et al., 2007; Reitsma et al., 2009; Trikalinos and Balion, 2012). They include, for example, combining several references to yield a single, better criterion. However, usually in these procedures it is assumed that the researchers already know whether their reference is GS or IR.

We have proposed a procedure which satisfies the need for distinguishing between two different assessment scenarios: perfect reference (GS) vs. imperfect reference (IR). Nested multinomial tree models built with the parameters that define those two models are fit. The two examples described show that sometimes it is better to assume that the reference is a GS (knowing that its reliability is not perfect, but virtually optimal), but at other times the suboptimality of the reference must be acknowledged. The identification of a reference as a gold standard or as an imperfect reference allows a better evidence-based choice of the better test available for a specific screening process.

An alternative to the approach taken here could be useful when there are no independent and homogeneous studies available, but there is a single study with a large sample. This alternative consists of a random partition of the sample into multiple segments. Although problems may arise due to the sub-samples are extracted from exactly the same large sample, this alternative should be evaluated in future simulation studies.

Psychometric meta-analysis (Vacha-Haase, 1998; Hunter and Schmidt, 2004; Rodriguez and Maeda, 2006; Botella et al., 2010) provides combined estimates of the psychometric properties of the data obtained with a given test. The procedure outlined in this paper can be applied to refine meta-analytic estimates of the accuracy of tests employed for binary classifications. Such meta-analyses integrate primary studies that assess the accuracy of binary classifications performed on a specific test (e.g., Botella et al., 2013). If the primary studies included in a meta-analysis have been carried out using an IR, the combined estimate of the accuracy will be incorrect, unless the imperfection of the reference is detected and assessed. The present procedure allows the detection of such suboptimal performance.

A limitation of the procedure is that it is not yet capable of managing situations where the classifications provided by the test are not conditionally independent of the classification provided by the reference.

### Conflict of interest statement

The authors declare that the research was conducted in the absence of any commercial or financial relationships that could be construed as a potential conflict of interest.
